# Analysis of Secure Apps for Daily Clinical Use by German Orthopedic Surgeons: Searching for the "Needle in a Haystack"

**DOI:** 10.2196/17085

**Published:** 2020-05-07

**Authors:** Florian Dittrich, Sascha Beck, Anna Katharina Harren, Felix Reinecke, Sebastian Serong, Jochen Jung, David Alexander Back, Milan Wolf, Stefan Landgraeber

**Affiliations:** 1 Department for Orthopaedics and Orthopaedics Surgery Saarland University Medical Center and Saarland University Faculty of Medicine Homburg Germany; 2 Sportsclinic Hellersen Lüdenscheid Germany; 3 Department of Plastic, Reconstructive & Aesthetic Surgery Specialized Clinic Hornheide Münster Germany; 4 Center for Orthopedics and Traumatology University Medicine Essen Essen Germany; 5 Department for Orthopedics Diakonie Hospital Bad Kreuznach Bad Kreuznach Germany; 6 Clinic of Traumatology and Orthopedics Bundeswehr Hospital Berlin Berlin Germany

**Keywords:** smartphone, mHealth, app, orthopedics, app store, screening

## Abstract

**Background:**

It is undeniable that appropriate smartphone apps offer enormous opportunities for dealing with future challenges in orthopedic surgery and public health, in general. However, it is still unclear how the apps currently available in the two major app stores can be used in daily clinical routine by German orthopedic surgeons.

**Objective:**

This study aimed to gain evidence regarding the quantity and quality of apps available in the two major app stores and their suitability for use by orthopedic surgeons in Germany.

**Methods:**

We conducted a systematic, keyword-based app store screening to obtain evidence concerning the quantity and quality of commercially available apps. Apps that met the inclusion criteria were evaluated using the *app synopsis–checklist for users* and the German Mobile App Rating Scale for secure use, trustworthiness, and quality.

**Results:**

The investigation revealed serious shortcomings regarding legal and medical aspects. Furthermore, most apps turned out to be useless and unsuitable for the clinical field of application (4242/4249, 99.84%). Finally, 7 trustworthy and high-quality apps (7/4249, 0.16%) offering secure usage in the daily clinical routine of orthopedists were identified. These apps mainly focused on education (5/7). None of them were CE (Conformité Européenne) certified. Moreover, there are no studies providing evidence that these apps have any positive use whatsoever.

**Conclusions:**

The data obtained in our study suggest that the number of trustworthy and high-quality apps on offer is extremely low. Nowadays, finding appropriate apps in the fast-moving, complex, dynamic, and rudimentarily controlled app stores is most challenging. Promising approaches, for example, systematic app store screenings, app-rating developments, reviews or app libraries, and the creation of consistent standards have been established. However, further efforts are necessary to ensure that these innovative mobile health apps not only provide the correct information but are also safe to use in daily clinical practice.

## Introduction

### Smartphone and Apps

It is only 12 years since smartphones started their triumphant progress through the world of communication media. Nowadays, daily smartphone usage for communication, collection of information, or data for private or professional purposes has become commonplace [1]. The portability and omnipresent accessibility of smartphones enable their usage anywhere and at any time [2]. After initial groundbreaking steps (including the E-Health Act of 2015) [3], the legislature recently gained considerable momentum in the direction of a stringent national digitization strategy. The “Law for better care through digitization and innovation” (Digitale-Versorgung-Gesetz) passed by the Bundestag on November 7, 2019, paved the way for the prescription of apps, the improved use of web video consultation services, and greater data security in the communication of health data [4].

Colloquially known as “apps,” mobile apps are defined as application software for mobile-operating systems. They are tailored to the users’ individual requirements, bring the smartphone to life, and thus, unleash the full potential of this new technology. These apps usually provide their application-specific functions via an intuitive user interface (“frontend”), specifically adapted to the mobile form factor, and often make use of web resources as well.

It is essential to differentiate between apps developed for patients and those intended for use by medical staff. Apart from apps that provide purely lifestyle advice, there are apps that can directly influence diagnosis or therapy of diseases and, therefore, should be regarded as medical devices [5]. However, there is no standard definition for apps in a medical context, which would enable users to differentiate between “lifestyle apps,” “health apps,” “medical apps,” or “care apps” [6].

Nowadays, apps can also be technically differentiated into native or web apps, each with advantages and disadvantages. Native apps are installed locally on the smartphone and make use of native application programming interfaces, which often leads to a significantly better performance and adaptation to the native look and feel of the respective platform. Web apps are webpages that have been optimized for running on mobile devices. Hybrid apps are based on web technologies but are packed as native apps and, therefore, have an intermediate role.

### App Stores

Apps can be bought and downloaded via several app stores. The major stores are the Google Play Store (Google LLC) and the App Store (Apple Inc). The simple distribution, low development costs, and the ease of use lead to a constantly changing and unmanageable supply. Owing to their complexity and rudimentarily regulated organization, the app stores’ offers are nontransparent and heterogeneous [7]. The range is so dynamic that the quantity and quality of apps can vary even from day to day [7,8]. With the rapid development of a fast-moving app industry, the number of apps available in the stores has exploded in the last decade. For instance, the number of apps offered in the “medical” category of the Google Play Store in October 2019 amounted to 42,989 [9].

Owing to inadequate legal, ethical, and medical regulations, many innovative apps operate in a grey area [10]. Recent data scandals have led to a basic distrust of mobile software that could be misused in the context of “big data” [11]. However, comprehensive information on app specifications, which is essential for safe usage in the medical context, is only provided sporadically in the app stores [7,10]. Inadequate store descriptions that provide no transparency and only insufficient information on the intended purpose and limitations of the apps as well as data privacy make it difficult to identify apps designed for the specific requirements of orthopedic surgeons. In addition, specific search terms and keywords are required to find an appropriate app [7]. However, even if there are a large number of matches, only a limited number of results are commonly displayed on the search interfaces provided by the store, and little is known about the criteria and algorithms based on which apps are selected and which of these are listed more prominently [12].

### Mobile Health in Orthopedic Surgeons Clinical Use

Irrespective of rapid developments in the field of medical apps, the information behavior of young physicians has fundamentally changed in recent years [13]. Numerous studies have focused on the potential benefits and consequences of smartphone usage in the fields of orthopedic surgery as a result of the rapidly growing mobile health (mHealth) implementation [14-18]. Currently, there are some app store–based screening reviews of commercially available apps that have been developed to address daily clinical issues in orthopedics [19,20]. These focus on spine surgery [21] or sports medicine [22].

However, to our knowledge, no studies have evaluated the availability and usability of apps directed at the specific needs of German orthopedic surgeons so far. mHealth apps that have not been developed for the German market are dubious from a legal and medical point of view if the algorithms and guidelines used do not comply with German requirements [23].

To address this gap, we conducted a systematic review for quantity and quality of commercially available German apps intended for use in everyday clinical routine in orthopedic surgery practices in Germany.

## Methods

### Systematic App Store–Screening Method

Appropriate apps were identified in a well-established, standardized, keyword-based, and systematic web search in the world’s largest web platforms for apps—the Google Play Store and the App Store [24]. The search took place between March 1 and April 27, 2019. For the search in the app stores, 23 German keywords were screened in all categories (Textbox 1). The search terms, which had a clear relation to the question, were defined by a group of 5 experts before initiating the study.

Two raters independently screened the Google Play Store and the App Store for each individual search term on the day of search. All apps that met the inclusion criteria were placed in individual “My Wish Lists” or Excel spreadsheets (Microsoft Corp).

**Figure 1:**
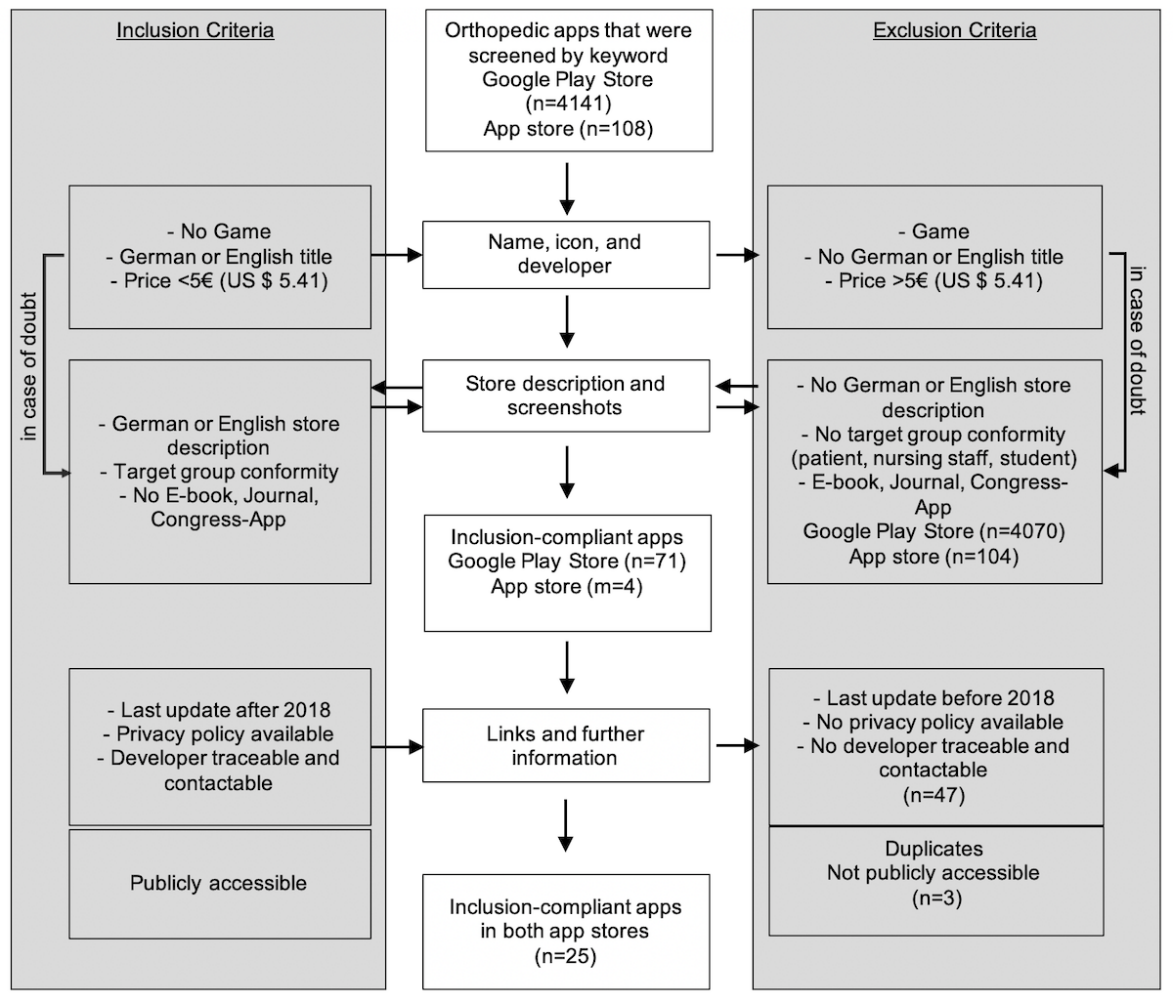
Flowchart screening process. E-book: electronic book.

For the purpose of standardizing the store search, the screening procedure was divided into the following 3 steps (Figure 1):

1. The first step involved a keyword search of apps in the context of orthopedics. The search term “orthopaedic” was always part of the search and was used alone or in combination with the other search terms (Textbox 1) in the Google Play Store and the App Store. The names, icons, and developers of the apps were checked against a priori defined inclusion and exclusion criteria (Figure 1). Apps were excluded if the icon, app name, or developer (1) clearly suggested a game, (2) no German or English name was chosen, and (3) the full use of the app exceeded a price of 5€ (US $5.41) per download.

If an app could not be clearly evaluated based on the overview page, the defined inclusion or exclusion criteria were applied to the store description mentioned in the detailed store view. If it was not possible to clearly differentiate in the detailed store view whether the inclusion criteria were met, the app temporarily remained in the study.

2. All apps deposited by the two examiners in their individual “My Wish List” (Google Play Store) or Excel table (App Store) were then re-evaluated by a 5-member group of experts for the existence of the abovementioned inclusion criteria and entered into single lists per app store. If there was no consensus, the app was still included in the study. If a full and light version of the same app was available, the light version was excluded.

3. If the exclusion criteria were not met, the store descriptions and screenshots were evaluated according to the existence of (1) a German- or English-language store description and (2) orthopedic-specific target group conformity. If the inclusion criteria were met, the apps remained in the study. Apps were excluded if their content was focused on topics that (1) were of no interest to the target group (eg, patients, students, nursing staff, and physiotherapists), (2) were not relevant for clinical orthopedic work (eg, promotion, electronic book [e-book], journal, or congress app), or (3) required external devices for use (eg, accelerometer-based activity monitoring). Further reasons for exclusion were if (4) no developer was identified, (5) no privacy statement was available, or (6) the store description was written in a language other than English or German.

4. The final step tested for (1) the existence of a German- or English-language data protection declaration as well as (2) the identification of the developer and (3) the time of the most recent update (2018 or 2019). Apps that were not updated in at least the previous year were removed.

After duplicates (identical apps found in the Google Play Store and the App Store) had been identified and excluded, the remaining apps, store descriptions, and links to the corresponding app store page were finally merged into one Excel table. If required, we paid for the full version of the app. If there were discrepancies between the two raters, consensus was again reached in the expert group.

**Original keywords used in the search and the resulting number of hits in the Google Play Store and App Store.**

“Orthopädie” (GPS: 249, AS: 53) OR “Orthopädie” AND “Untersuchung” (GPS: 186, AS: 1) OR “Orthopädie” AND “Untersuchungstechniken” (GPS: 154, AS: 0) OR “Orthopädie” AND “Röntgen” (GPS: 249, AS: 1) OR “Orthopädie” AND “Bildgebung” (GPS: 217, AS: 0) OR “Orthopädie” AND “Operation” (GPS: 249, AS: 0) OR “Orthopädie” AND “Operationstechnik” (GPS: 214, AS: 0) OR “Orthopädie” AND “operativer Zugangsweg” (GPS: 180, AS: 0) OR “Orthopädie” AND “Operationsanleitung” (GPS: 103, AS: 0) OR “Orthopädie” AND “Implantat” (GPS: 248, AS: 0) OR “Orthopädie” AND “Rehabilitation” (GPS: 248, AS: 1) OR “Orthopädie” AND “Nachsorge” (GPS: 124, AS: 0) OR “Orthopädie” AND “Therapie” (GPS: 249, AS: 5) OR “Orthopädie” AND “Diagnose” (GPS: 246, AS: 2) OR “Orthopädie” AND “Diagnostik” (GPS: 247, AS: 1) OR “Orthopädie” AND “Leitlinie” (GPS: 117, AS: 0) OR “Orthopädie” AND “Endoprothetik” (GPS: 122, AS: 9) OR “Orthopädie” AND “Lagerung” (GPS: 111, AS: 0) OR “Orthopädie” AND “Mikrobiologie” (GPS: 122, AS: 0) OR “Orthopädie” AND “Wirbelsäule” (GPS: 161, AS: 20) OR “Orthopädie” AND “Skoliose” (GPS: 106, AS: 2) OR “Orthopädie” AND “Schmerztherapie” (GPS: 112, AS: 13) OR “Orthopädie” AND “Klassifikationen” (GPS: 127, AS: 0)

### Specific App Ranking

Apps that met all inclusion criteria were evaluated using the *app synopsis–checklist for users* [25-27] regarding secure use and trustworthiness in the context of German data protection regulations. Appropriate apps were downloaded, installed, and evaluated from May to July 2019 by 5 raters (FD, DB, KH, FR, and SS) on various smartphones with Android and iPhone operating systems (Samsung Galaxy S8, iPhone 7, and iPhone 8). The evaluators ran all apps on their smartphones for at least 10 days to review all app features and extract data about app features or additional functions.

In a second step, trustworthy apps were rated and ranked using the “German Mobile App Rating Scale” (MARS-G) [28].

All investigations on humans were carried out with the consent of the responsible ethics committee in accordance with the national law and the Declaration of Helsinki of 1975 (in the current, revised version).

### App Synopsis

The app synopsis is a well-established tool for evaluation of the quality and trustworthiness of apps intended for use in Germany [25,26]. It was developed by the Peter L. Reichertz Institute for Medical Informatics at the Hannover Medical School with special focus on the guidelines and regulations applicable in Germany. The *app synopsis* enables app users without a professional information technology background to estimate the trustworthiness of apps. Questions regarding the 8 sections *medical device*, *intended purpose*, *functionality*, *scientific quality*, *restrictions and limits*, *risks*, *reliability of content*, and *data protection* must be marked with one of the following 3 options: “yes,” “no“, or “unclear.” Some, but not all, answer qualities have a higher relevance in the context of an app’s trustworthiness, and the answer options are therefore highlighted based on a signal light system. The better the trustworthiness of an app, the more “green” markers, that is, “yes” answers, it should have obtained. A field highlighted in “red” is an indication for reasonable skepticism about the trustworthiness of an app regarding the respective criterion. If, on the other hand, only fields with a “green” background have been marked, this is an indication of higher trustworthiness compared with apps with fewer or only single positive answers. “Orange” ratings may still indicate trustworthiness for an app, albeit to a lesser extent, and they should be used with caution [27].

### German Mobile App Rating Scale

The MARS-G was developed for professionals to rate app quality and includes the sections *classification*, *quality*, *satisfaction*, and a modifiable app-specific section. The MARS rating is a well-established assessment scale for medical app quality [28]. MARS-G items are scored using a 5-point Likert scale (1=inadequate, 2=poor, 3=acceptable, 4=good, and 5=excellent).

The *classification* section provides descriptive information about the app. The *objective app quality* section includes 19 items divided into 4 subscales, namely *engagement*, *functionality*, *aesthetics*, and *information quality*, and further 10 items comprising 2 subjective subscales, namely *subjective app quality* and *perceived impact*.

The *subjective quality* section contains 4 items evaluating the user’s overall satisfaction. The 4 sections of the English MARS version were expanded in the MARS-G by an additional section focusing on the *medical gain* of an app. The 5 subscales and the overall score determine the app’s quality [29]. Five reviewers (FD, DB, KH, SS, and FR) watched the associated MARS-G instructional video about how to use the MARS-G scale before rating [30].

### Data Analysis

The paper-based app synopsis and the MARS-G were converted into a digital questionnaire on the *Google Docs* platform (Google LLC). Five reviewers rated the “AO/OTA Fracture Classification” app to evaluate interrater reliability and 8 to 10 randomly selected apps. Data were saved and then transferred to an Excel table. Descriptive statistics were calculated for all items. The interclass correlation coefficients (ICCs) were calculated between the 5 reviewers. We selected an individual absolute agreement intraclass correlation (AA-ICC) for a two-way mixed model on the basis of ICC guidelines by Shrout and Fleiss [31]. The interpretation for ICC interrater agreement measures followed the guidelines of Koo et al [32]. All statistical analyses were conducted using SPSS (version 25, IBM Corp).

## Results

**Figure 2:**
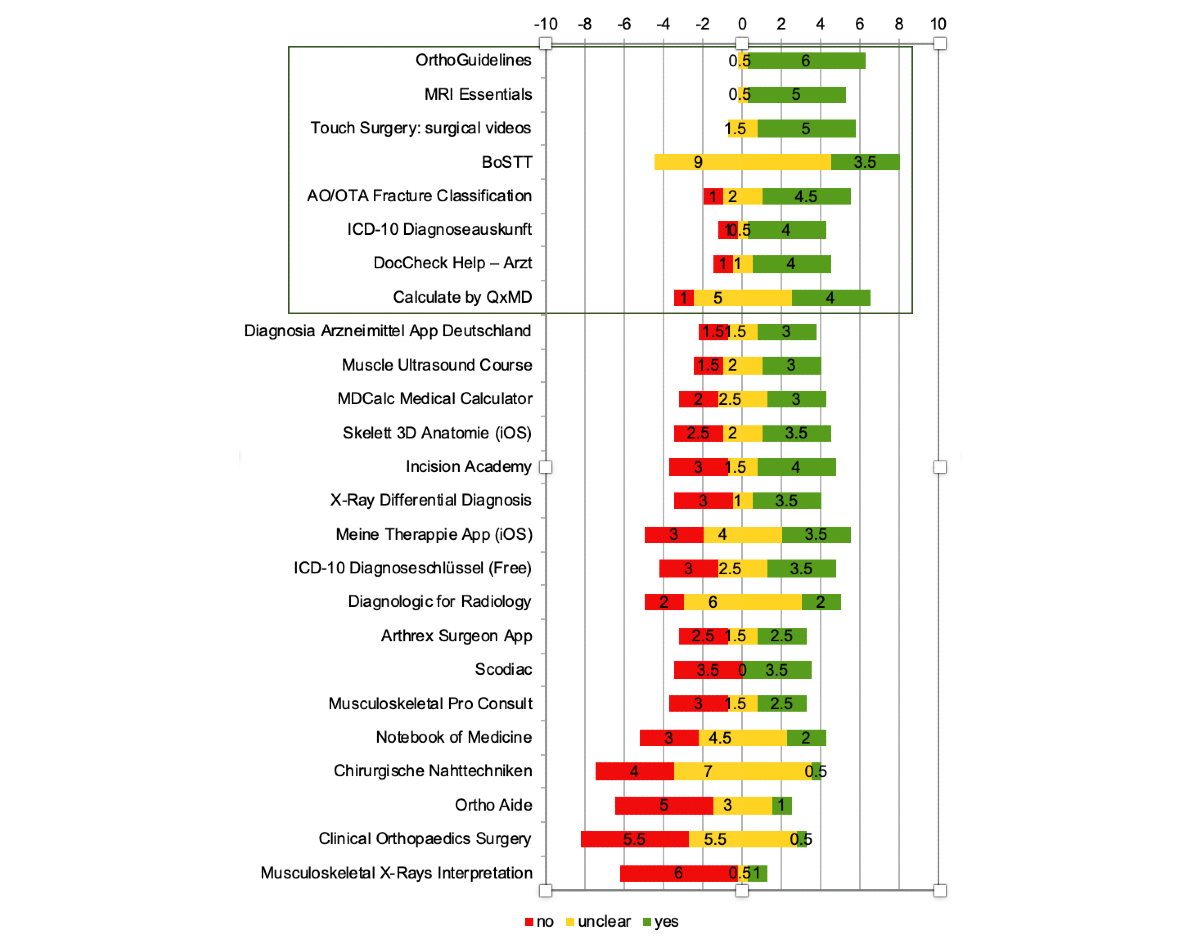
Mean ratings for trusted apps using the app synopsis—checklist for users. Every single app was ranked by two raters. Apps were primarily ranked in a “trustworthiness scale” (yes=+, unclear=±, and no=−) and secondarily by the following criteria: (1) ascending red (missing), (2) descending green (existing), and (3) ascending orange (unclear) trustworthiness marker. The lower red, higher green, and lower orange markers’ quantity, the higher the app’s trustworthiness. AO/OTA: Arbeitsgemeinschaft für Osteosynthesefragen/Orthopedic Trauma Association; BoSTT: bone and soft tissue tumors-case studies; ICD-10: International classification of diseases, tenth revision; MRI: magnetic resonance imaging.

Our systematic web search revealed 4141 hits in the Google Play Store and 108 hits in the App Store using the aforementioned 23 keywords. After evaluating the publicly available information, 1.71% (71/4141) of Google Play Store–screened and 3.7% (4/108) of App Store–screened apps met the formal inclusion criteria. Finally, 0.59% (25/4249) of apps met the minimum requirements of data protection regulation (Figure 1). These apps were downloaded and evaluated using the app synopsis. None of the apps were CE certified. Of these, 8 apps were classified as trustworthy. Good interrater reliability (two-way mixed model single measure AA-ICC=0.78, 95% CI 0.68-0.86) was shown following the guidelines for ICC interpretation established by Koo et al [30]. No trustworthiness markers were missing for the apps *OrthoGuidelines*, *MRI Essentials*, *Touch Surgery: surgical videos*, and *BOSTT*. Another 4 apps, *ICD-10 Diagnoseauskunft*, *DocCheck Help – Arzt*, *AO/OTA Fracture Classification*, and *Calculate by QxMD*, lacked only 1 trust marker on average. Apps that lacked more than one marker on average were classified “not trustworthy.” Therefore, concerns had to be raised about the trustworthiness and transparency of the remaining 17 apps that lacked 3.21 (SD 1.26) trustworthiness markers on average (Figure 2).

**Figure 3:**
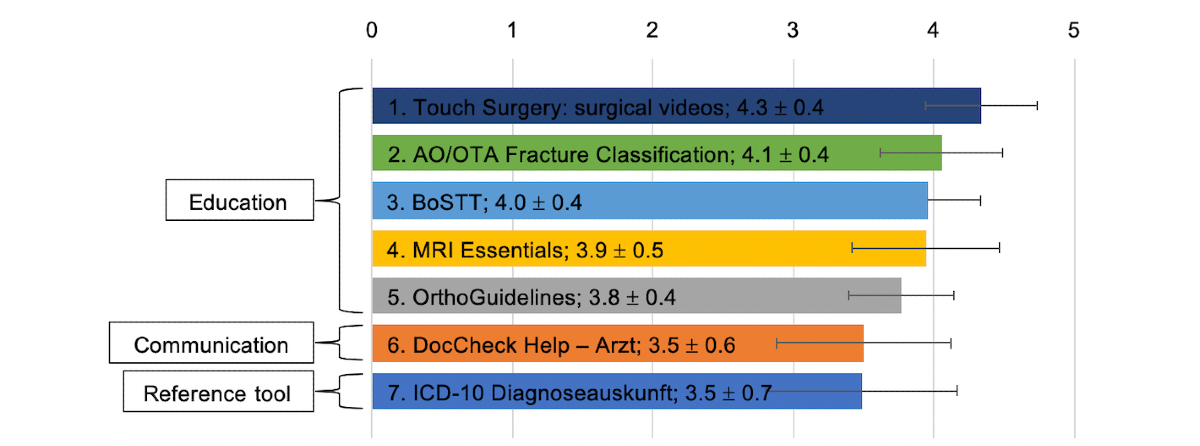
Mean overall rating for trusted apps using the “MARS-G”. Every single app was ranked by two raters. AO/OTA: Arbeitsgemeinschaft für Osteosynthesefragen/Orthopedic Trauma Association; BoSTT: bone and soft tissue tumors-case studies; ICD-10: International classification of diseases, tenth revision; MRI: magnetic resonance imaging; MARS-G: German Mobile App Rating Scale.

**Figure 4:**
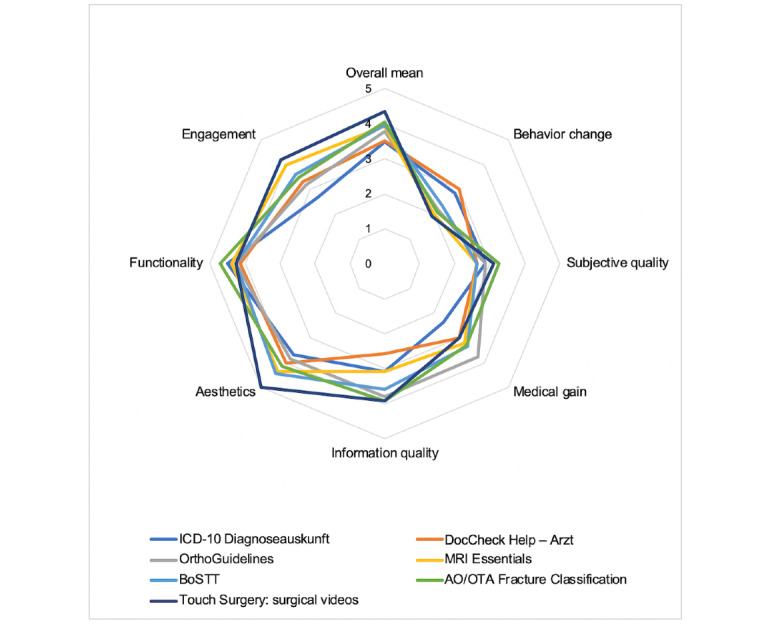
Mean section ratings for trusted apps using the “MARS-G”. Every single app was ranked by two raters. AO/OTA: Arbeitsgemeinschaft für Osteosynthesefragen/Orthopedic Trauma Association; BoSTT: bone and soft tissue tumors-case studies; ICD-10: International classification of diseases, tenth revision; MRI: magnetic resonance imaging; MARS-G: German Mobile App Rating Scale.

Subsequently, the quality of the trustworthy apps was evaluated using the MARS-G. The *Calculate by QxMD* app failed to launch at the time of the MARS-G rating and was, therefore, excluded. Moderate interrater reliability (two-way mixed model, single measure AA-ICC=0.58, 95% CI 0.43-0.74) was shown for the MARS-G rating. MARS-G rating revealed the highest overall mean score with 4.3 (SD 0.4) for the app *Touch Surgery: surgical videos* and the lowest score with 3.5 (SD 0.7) for the app *ICD-10 Diagnoseauskunft* (Figures 3 and 4).

## Discussion

### Principal Findings

The two major app stores were browsed for apps intended for use in everyday clinical routine of orthopedic surgeons. On the basis of keywords, 4249 apps were detected. These were evaluated for quality, safety, and usability. Most of these apps were considered inappropriate for use in daily clinical practice (eg, Games and e-books; 4242/4249, 99.84%). To find an appropriate app, an average of 607 (4249/7) apps had to be screened. Finally, 0.16% (7/4249) of apps were considered reliable, secure, and of high quality in the app synopsis and MARS-G analyses. Interestingly enough, apps that achieved a high score in the app synopsis also received a good evaluation in MARS testing. Nevertheless, none of these apps were CE certified nor had their purpose been evaluated in studies. The remaining apps focused on educational (5/7), communicative (1/7), and reference (1/7) aspects.

The identification of apps tailored to the specific needs of orthopedic surgeons was hampered by a lack of transparency, inadequate store descriptions, missing information on limitations of the app, or lacking precise and public declaration regarding the intended purpose and data protection. Nontransparent store descriptions and missing app-related meta-information represent a great challenge for users when trying to find their way around the app market [12]. Moreover, a systematic evaluation of the top-ranked mental health apps’ store descriptions identified the use of scientific language as the most frequently employed strategy to suggest effectiveness [33]. In many cases, the detection of appropriate apps is only successful if specific search terms are used [7]. If the search results in a large number of matches, store providers select a limited number of apps without naming the selection criteria used [12]. This aspect harbors the risk that users will find unsuitable apps and smartphone apps or fail to identify suitable apps, as these may not be displayed at all. One example of inappropriate app usage is the use of *WhatsApp* (Facebook Inc) as a communication tool in everyday clinical practice. Using *WhatsApp* to send patient-related data is not safe and does not comply with the EU-DSGVO regulations because the mobile phone address book is extracted regularly. Messages are already sent in encoded form end-to-end, but the metadata readout is not affected [34].

### Limitations

The presented work has some limitations. The matches using the aforementioned keywords for search in the Google Play Store and the App Store only refer to a priori defined German search words in their nominal form. Moreover, it was not possible to determine how the search result might be influenced by an adjustment of the search terms and the combination of individual words because the underlying app stores’ algorithms remain unclear. To ensure a transparent and objective systematic app store search, already well-established methods were applied [24,35,36]. However, none of these methods have been sufficiently validated so far. This must be addressed by future studies.

A further limiting factor is the fact that the apps included in the study were technically and conceptually extremely inhomogeneous, resulting in a great diversity of application areas and legal aspects. Therefore, a discussion is needed as to whether proper evaluation of such a collection of apps is possible with only one standard rating tool. This is underlined by the moderate interrater reliability using MARS-G rating, suggesting that the score itself is rather subjective. These findings are in line with actual systematic app ratings including more than two raters [24,35]. The combination of existing scores might be valuable [36].

### Outlook

Owing to the fast-moving, complex, dynamic, and rudimentarily controlled nature of the app stores, the market is heterogeneous and not transparent for the user on the one hand [7,8], but on the other hand, it might become a highly productive innovation incubator. Therefore, the current situation should be seen both as an opportunity and a risk. Identifying high-quality apps among the wide range of apps currently on the market represents a great challenge. It is like trying to find the famous needle in a haystack. However, some approaches have already addressed this issue:

1. A growing number of publications are critically addressing the currently available apps and have conducted manual systematic app store searches [24,35,36]. These publications may serve as a basis for content and methodological approaches to personal app searches. However, this method is very time consuming. Newly developed semiautomated search methods are based on filtering processes using predefined criteria, for example, the semiautomated retrospective App Store analysis, and might be extended with algorithmic analysis or artificial intelligence in the future [12].
2. By developing the MARS, a first attempt was made to create a tool dedicated to an objective assessment of the app’s content and technical specifications, which is essential to enable a comparison between apps [28]. The app synopsis, also used in this study, primarily focuses on an app’s trustworthiness. Nevertheless, apps that collect and process sensitive patient-related data must fulfill higher data protection requirements than apps that are used for coding purposes and do not collect data at all. But evaluation with a standard-based tool might lead to a false-negative rating of the coding app. Therefore, the existing ratings have been constantly improved, and new, more specific tools have been developed [29,37]. An increase in app rating quality might be achieved if adaptive ratings focusing on the intended app purpose were developed.
3. Several professional associations as well as private institutions aim to review apps and publish them in app libraries. The
  NHS Apps Library
  only recommends safe and secure apps in the United Kingdom. Developers must answer a standardized, transparent, and publicly available range of digital assessment questions designed by experts from technical and policy backgrounds [38]. For mental health apps, a nonprofit organization, in cooperation with several universities, provides guidelines and app reviews on the webpage PsyberGuide. The standardized review process is based on credibility, user experience, and transparency using established rating tools [39]. In the field of orthopedic surgery, the private webpage
  TopOrthoApps
  gives app information and reviews, though the review process is not transparent and the studies presented seem outdated [40].

In the absence of consistent legal, ethical, and medical regulations, numerous innovative apps remain in a grey area and struggle to deploy their full potential. In times when international technology concerns are already optimizing innovative technologies (eg, the use of artificial intelligence) in apps, the framework conditions for a solid but also dynamic and adaptive mHealth strategy must be developed in the German health care system.

### Conclusions

The benefits of the appropriate use of smartphones and apps in the field of orthopedic surgery are undeniable and have enormous potential for dealing with future challenges in public health. The data gained in our study suggest that the number of trustworthy and high-quality apps on offer is extremely low. Most of the apps display serious shortcomings regarding legal and medical aspects. The fast-moving, complex, and dynamic nature of the app stores, which are under only rudimentary control, harbors the risk of inappropriate app usage. However, the stores also provide important innovations in health care. The search for the appropriate app is a considerable challenge. Promising approaches, for example, systematic app store screenings, app rating developments, reviews or app libraries, and the creation of consistent standards have already been established. Further efforts and interdisciplinary cooperation are required to detect innovative mHealth solutions that can be utilized in a safe and secure manner in the work of orthopedic surgeons.

## Abbreviations

AA-ICC: absolute agreement intraclass correlation
ICC: interclass correlation coefficients
MARS-G: German Mobile App Rating Scale
mHealth: mobile health
