# Correction: Analysis of Secure Apps for Daily Clinical Use by German Orthopedic Surgeons: Searching for the “Needle in a Haystack”

**DOI:** 10.2196/21600

**Published:** 2020-06-24

**Authors:** Florian Dittrich, Sascha Beck, Anna Katharina Harren, Felix Reinecke, Sebastian Serong, Jochen Jung, David Alexander Back, Milan Wolf, Stefan Landgraeber

**Affiliations:** 1 Department for Orthopaedics and Orthopaedics Surgery Saarland University Medical Center and Saarland University Faculty of Medicine Homburg Germany; 2 Sportsclinic Hellersen Lüdenscheid Germany; 3 Department of Plastic, Reconstructive & Aesthetic Surgery Specialized Clinic Hornheide Münster Germany; 4 Center for Orthopedics and Traumatology University Medicine Essen Essen Germany; 5 Department for Orthopedics Diakonie Hospital Bad Kreuznach Bad Kreuznach Germany; 6 Clinic of Traumatology and Orthopedics Bundeswehr Hospital Berlin Berlin Germany

In “Analysis of Secure Apps for Daily Clinical Use by German Orthopedic Surgeons: Searching for the “Needle in a Haystack” (JMIR Mhealth Uhealth 2020;8(5):e17085) the authors noted an error.

The paper was incorrectly listed as the article type “Review.” The correct article type is “Original Paper.”

The correction will appear in the online version of the paper on the JMIR Publications website on June 24, 2020, together with the publication of this correction notice. Because this was made after submission to PubMed, PubMed Central, and other full-text repositories, the corrected article has also been resubmitted to those repositories.

